# CD19嵌合抗原受体T细胞治疗病灶大于7.5 cm的复发/难治B细胞淋巴瘤肿瘤局部反应与疗效分析

**DOI:** 10.3760/cma.j.issn.0253-2727.2021.07.007

**Published:** 2021-07

**Authors:** 青 李, 昊彬 邓, 美静 刘, 翠翠 吕, 海波 朱, 嘉 王, 怡丽 蒋, 业迪 蒲, 嫣雨 江, 维 李, 琦 邓

**Affiliations:** 1 天津市第一中心医院血液科，南开大学医学院 300192 Department of Hematology, Tianjin First Central Hospital, School of Medicine, Nankai University, Tianjin 300192, China; 2 天津医科大学一中心临床学院 300070 The First Central Clinical College of Tianjin Medical University, Tianjin 300070, China; 3 天津医科大学肿瘤医院淋巴瘤科，中美淋巴瘤血液诊治中心，国家肿瘤临床医学研究中心，天津市肿瘤防治重点实验室，天津市恶性肿瘤临床医学研究中心 300060 Department of Lymphoma, Tianjin Medical University Cancer Institute and Hospital, National Clinical Research Center of Cancer, Key Laboratory of Cancer Prevention and Therapy, Tianjin's Clinical Research Center for Cancer, the Sino-US Center for Lymphoma and Leukemia Research, Tianjin 300060, China

**Keywords:** 复发/难治, 非霍奇金淋巴瘤, 嵌合抗原受体T细胞, 不良反应, 疗效, Relapse/refractory, Non-Hodgkin's lymphoma, CD19 Chimeric antigen receptor T cell, Side effects, Efficacy

## Abstract

**目的:**

观察病灶>7.5 cm的复发/难治B细胞非霍奇金淋巴瘤（R/R NHL）患者CD19嵌合抗原受体T细胞（CAR-T细胞）治疗的肿瘤局部反应及疗效。

**方法:**

以2018年8月至2020年5月接受CD19 CAR-T细胞治疗的病灶>7.5 cm的32例R/R NHL患者为研究对象，流式细胞仪检测CD19CAR-T细胞的体内扩增情况；酶联免疫吸附测定法检测患者外周血中细胞因子水平；观察全身不良反应及肿瘤局部反应，分析总有效率（ORR）及总生存（OS）情况。

**结果:**

① 32例患者CAR-T细胞治疗后，13例获得完全缓解（CR）（40.63％），10例获得部分缓解（PR）（31.25％），ORR为71.88％。② 23例有效患者均发生细胞因子释放综合征（CRS），其中1～2级13例，3～4级10例；而疾病稳定+疾病进展（SD+PD）组9例患者CRS均为1～2级（*P*＝0.030）。③共15例（46.9％）患者发生肿瘤局部反应，其中CR 9例、PR 5例、SD 1例，肿瘤局部反应包括：浅表肿物直径增大且伴红肿热痛；深部肿物表现为腹痛、腹胀、憋气以及肿瘤局部疼痛、烧灼，瘤体增大或伴局部水肿；肿瘤局部出现渗出性病变，可见于腹腔、胸膜腔等。④有效组CD19 CAR-T细胞峰值高于SD+PD组［16.8％（5.3％～48.2％）对2.9％（1.5％～5.7％），*z*＝−4.297，*P*<0.001］，有效组中出现肿瘤局部反应患者CD19 CAR-T细胞峰值高于未出现肿瘤局部反应患者［22.2％（10.5％～48.2％）对12.6％（5.3％～21.6％），*z*＝−3.213，*P*＝0.001］，多发肿块组CD19 CAR-T细胞峰值高于单发肿块组［35.8％（1.5％～48.2％）对16.8％（10.5％～18.5％），*z*＝−2.023，*P*＝0.040］。⑤肿瘤局部反应出现和瘤体缩小时间，均较全身不良反应时间延迟。⑥有效患者中出现肿瘤局部反应者OS率高于未出现肿瘤局部反应者，但差异无统计学意义（75.0％对34.6％，*P*＝0.169）。

**结论:**

病灶>7.5 cm的R/R NHL患者CD19 CAR-T细胞治疗，近一半出现肿瘤局部反应，发生时间迟于全身不良反应开始的时间。**临床试验注册：**中国临床试验注册中心（ChiCTR1800018059）

## Abstract (en)

**Objective:**

To observe the local reactions and efficacy of CD19 CAR-T therapy in recurrence/refractory B-cell non-Hodgkin's lymphoma (R/R NHL) patients with >7.5 cm lesions.

**Methods:**

32 R/R NHL patients with >7.5 cm lesions were enrolled and injected with CD19 CAR-T cells. Flow cytometry was used to detect and observe the amplification of CD19 CAR-T cells in vivo. Enzyme-linked immunosorbent assay (ELISA) was used to detect cytokines in peripheral blood of patients. The side effects of CD19 CAR-T cell therapy included systemic side effects and local reactions of tumor. The local side effects were observed by Ultrasound, Computed tomography and Magnetic resonance imaging. Treatment options included glucocorticoid, interleukin-6 antibody and drainage of exudate. Overall response rate (ORR) and overall survival rate (OS) were observed.

**Results:**

①Among the 32 patients, CR (40.63％), PR (31.25％) and ORR (71.88％) were 13, 10 and 23, respectively. ②In all 23 patients received ORR, 13 patients had grade 1–2 CRS, while 10 patients had grade 3–4 CRS. All the 9 patients in the SD+PD group had grade 1–2 CRS（*P*＝0.030）. ③A total of 15 patients with tumor local reactions, included 9 patients with CR, 5 patients with PR and 1 patient with SD. The local reactions of the tumor included that the diameter of the superficial lesions increased with redness, swelling and heat pain. The deep lesions presented abdominal pain, abdominal distension, suffocation and local pain，and burning of the tumor. The deep lesions were enlarged or accompanied by local edema. The local exudative lesions were found in the abdominal cavity and pleural cavity. ④ Peak proportion of CD19 CAR-T cells in ORR group was higher than that of in SD+PD group［16.8％（5.3％–48.2％）*vs* 2.9％（1.5％–5.7％）, *z*＝−4.297, *P*<0.001］. The peak proportion of CD19 CAR-T cells in ORR group with local reactions was higher than that of in patients without local reactions［22.2％（10.5％–48.2％）*vs* 12.6％（5.3％–21.6％）, *z*＝−3.213, *P*＝0.001］. The peak proportion of CD19 CAR-T cells in multiple lesion group was higher than that of in single lesion group［35.8％（1.5％–48.2％）*vs* 16.8％（10.5％–18.5％）, *z*＝−2.023, *P*＝0.040］. ⑤Occurrence of local reactions and tumor shrinkage time were both delayed compared with systemic side effects. ⑥In the ORR group, the OS of patients with tumor local reactions was longer than that of patients without tumor local reactions, but there was no difference in the two groups（75％ *vs* 34.6％, *P*＝0.169）.

**Conclusion:**

CD19 CAR-T cell therapy in R/R NHL patients with >7.5 cm lesions might cause tumor local reactions later than systemic side effects. Clinicaltrial: ChiCTR1800018059

CD19嵌合抗原受体T细胞（CAR-T细胞）治疗复发/难治（R/R）B细胞非霍奇金淋巴瘤（NHL）近年来取得了非常显著的疗效[1]–[4]。在R/R NHL患者中，CD19 CAR-T细胞治疗毒性既包括全身不良反应，也包括肿瘤的局部反应。细胞因子释放综合征（CRS）和CAR-T细胞相关脑病综合征（CRES）等不良反应，在高肿瘤负荷患者中可能具有致命性[5]。本研究中，我们总结本中心CD19 CAR-T细胞治疗32例淋巴瘤病灶>7.5 cm的R/R NHL患者的疗效和不良反应，排除原发病进展及感染因素，观察治疗过程中肿瘤局部出现的肿胀、疼痛及水肿等症状特征及其对预后的影响，现报道如下。

## 病例与方法

一、病例

2018年8月至2020年5月于我院接收的R/R NHL患者33例，所有患者均具有至少1个直径>7.5 cm可评估的淋巴瘤病灶。除外1例患者未达评价时间死亡（CAR-T细胞输注后7 d），其余32例入组本研究。收集32例患者入组时临床资料，包括性别、年龄、病理类型、Ann Arbor分期、入组时的淋巴瘤国际预后指数（IPI评分）、接受过的治疗线数、是否伴有双打击/双表达、肿块数量和最大直径（cm）等。随访截止时间为2020年10月30日。

二、CD19 CAR-T细胞制备

单采患者外周血单个核细胞制备CD19 CAR-T细胞，磁珠分选采集物中CD3^+^ T细胞，CD3/CD28磁珠扩增CD3^+^ T细胞，进而以含有IL-2、谷氨酰胺的T细胞专用培养基培养。培养第4天转染CD19 CAR慢病毒（第二代、人源化CD19 CAR慢病毒，上海吉倍公司提供），培养第5天采用流式细胞仪检测CD19 CAR-T细胞的转染效率，培养第12天收获CD19 CAR-T细胞并计数。

三、CD19 CAR-T细胞的输注

氟达拉滨30 mg·m^−2^·d^−1^（−4～−2 d）、环磷酰胺300 mg·m^−2^·d^−1^（−4～−3 d）进行淋巴细胞清除后，回输自体人源化CD19 CAR-T细胞2×10^6^/kg。所有患者未应用程序性死亡受体-1（PD-1）抑制剂。

四、CD19 CAR-T细胞的体内扩增及细胞因子水平检测

分别在CD19 CAR-T细胞输注前，输注后第4、7、14、21及28天，以流式细胞术检测患者外周血中CD19 CAR-T细胞的比例；部分患者肿瘤局部可以采集到液体等可进行流式细胞术检测的标本，进行肿瘤局部标本的CD19 CAR-T细胞比例检测。采用酶联免疫吸附测定法（ELISA）检测患者外周血中IL-6、IL-2R、IL-10及TNF-α的分泌水平。根据文献[6]分级标准进行CRS分级评定，根据文献[7]进行CRES分级评定。

五、肿瘤局部反应的临床表现与治疗对策

肿瘤大小及局部反应采用超声、CT、MRI以及临床可见肿块的直接测量等方法进行评估。肿瘤局部监测的时间点包括：①接受CD19 CAR-T细胞治疗前；②细胞治疗过程中；③细胞输注后1个月、2个月评价达到最大疗效的时间。

1. 浅表部位肿瘤局部反应：浅表肿物出现直径增大且伴红肿热痛症状。临床给予局部对症处理，无缓解或伴有明显全身症状患者给予糖皮质激素治疗。

2. 深部肿瘤局部反应：腹腔、纵隔等浅表不可触及的肿物，临床表现为腹痛、腹胀、憋气以及肿瘤局部疼痛、烧灼等症状。超声、CT或MRI检查可见肿瘤体积增大或伴局部水肿。临床对策为对症处理及临时给予糖皮质激素治疗（甲泼尼龙20～40 mg），影像学检查密切监测肿瘤变化，压迫症状明显者给予IL-6抗体（4 mg/kg），每天应用，至局部反应开始消退。

3. 肿瘤局部出现渗出性病变：部分患者肿瘤局部出现大量组织液渗出表现，临床表现为腹痛、腹胀、憋气、局部胀痛等，部位可见于腹腔、胸膜腔以及浅表肿物部位，超声、CT检查可明确诊断。局部渗出性病变明显者给予局部引流（胸腔引流、腹腔引流以及局部切开引流等）；同时全身给予对症、糖皮质激素及IL-6抗体治疗。此部分患者引流液送检流式进行CD19 CAR-T细胞检测。

六、疗效评价

在复发/难治NHL患者CD19 CAR-T细胞输注后30 d及60 d评估治疗反应，包括完全缓解（CR）、部分缓解（PR）、疾病稳定（SD）、疾病进展（PD）[8]，有效包括CR+PR。总生存（OS）定义为入组至死亡或随访截止时间。

七、CD19 CAR DNA表达水平的监测

采用定量PCR（qPCR）监测CD19 CAR DNA水平，比较不同疗效患者CD19 CAR DNA水平。

八、统计学处理

采用SPSS 26.0进行数据分析。计量资料采用中位数（范围）进行描述，组间比较采用Mann-Whitney *U*检验；分类变量组间比较采用精确概率法。采用Kaplan-Meier法绘制生存曲线，组间差异比较采用Log-rank检验。*P*<0.05为差异有统计学意义。

## 结果

一、一般资料

32例患者中，男25例，女7例，中位年龄53（32～77）岁。病理类型、分期、入组时的IPI评分及接受过的治疗线数、肿瘤数量和最大直径等临床资料见表1，所有患者无中枢神经系统淋巴瘤或淋巴瘤中枢浸润。

**表1:** 32例CD19嵌合抗原受体T细胞治疗复发/难治B细胞非霍奇金淋巴瘤患者临床资料

例号	性别	年龄（岁）	病理类型	AnnArbor分期	IPI评分	治疗线数	双打击/双表达	肿瘤数量（个）	肿瘤最大直径（cm）
1	女	71	non-GCB	Ⅳ	3	2	双打击	≥2	12.2
2	男	58	non-GCB	Ⅳ	5	3	无	≥2	23.2
3	男	52	non-GCB	Ⅲ	4	4	无	≥2	9.4
4	男	54	GCB	Ⅱ	2	2	双表达	1	8.5
5	男	73	non-GCB	Ⅱ	3	2	双表达	1	13.1
6	男	61	GCB	Ⅲ	4	3	双打击	≥2	13.5
7	男	51	GCB	Ⅲ	4	2	双打击	≥2	12.6
8	男	31	GCB	Ⅱ	2	2	双表达	≥2	7.6
9	女	39	non-GCB	Ⅳ	5	2	无	≥2	18.9
10	男	46	GCB	Ⅲ	3	3	无	1	15.3
11	男	42	GCB	Ⅳ	4	3	双打击	≥2	7.6
12	男	50	non-GCB	Ⅲ	4	3	双打击	≥2	8.9
13	男	63	non-GCB	Ⅱ	2	3	双表达	≥2	8.8
14	女	60	GCB	Ⅳ	3	4	无	1	7.9
15	男	68	non-GCB	Ⅳ	3	2	无	≥2	7.7
16	男	43	non-GCB	Ⅳ	5	2	双打击	≥2	7.9
17	男	77	non-GCB	Ⅲ	4	3	无	≥2	8.3
18	女	46	non-GCB	Ⅲ	4	4	双表达	≥2	16.9
19	男	33	non-GCB	Ⅱ	3	3	双表达	1	20.3
20	女	41	tFL	Ⅳ	4	2	无	≥2	8.8
21	女	52	non-GCB	Ⅳ	5	2	双打击	≥2	9.2
22	男	69	GCB	Ⅲ	4	2	双打击	≥2	15.8
23	男	59	non-GCB	Ⅲ	3	3	双表达	1	8.6
24	男	32	non-GCB	Ⅳ	5	3	双表达	≥2	8.1
25	男	42	non-GCB	Ⅲ	3	3	无	1	7.8
26	女	46	non-GCB	Ⅳ	5	4	双打击	≥2	23.5
27	男	48	non-GCB	Ⅲ	4	4	三打击	1	8.9
28	男	76	non-GCB	Ⅲ	3	3	无	1	11.7
29	男	50	non-GCB	Ⅳ	5	3	双表达	≥2	7.9
30	男	51	non-GCB	Ⅱ	3	2	无	≥2	8.3
31	男	62	non-GCB	Ⅳ	4	3	双打击	≥2	8.7
32	男	52	FL	Ⅳ	5	3	无	≥2	20.6

注：GCB：生发中心型；Non-GCB：非生发中心型；tFL：转化型滤泡淋巴瘤；FL：滤泡淋巴瘤；IPI：国际预后指数

二、CD19 CAR-T细胞的转染率和输注情况

CD19 CAR-T细胞的转染率为（52.15±15.66）％，培养成熟收获细胞计数为（8.35±3.04）×10^6^/kg，输注CD19 CAR-T细胞（2.03±0.29）×10^6^/kg。

三、CD19 CAR-T细胞治疗的疗效评价

32例患者中，CR 13例（40.63％），PR 10例（31.25％），SD 5例（15.62％），PD 4例（12.50％）；总有效率（ORR）为71.88％。

四、CD19 CAR-T细胞治疗中全身不良反应

在CD19 CAR-T细胞治疗过程中，患者出现发热伴寒战、心动过速、咳嗽、恶心、水肿、头痛等全身不良反应的临床表现（表2）。有效组23例患者中，CRS中1～2级13例（56.5％），3～4级10例（43.5％）；SD+PD患者9例，CRS均为1～2级，差异有统计学意义（*P*＝0.030）；无一例患者出现CRES。

**表2:** 32例复发/难治B细胞非霍奇金淋巴瘤患者CD19嵌合抗原受体T细胞治疗中全身不良反应

全身不良反应	有肿瘤局部反应（15例）	无肿瘤局部反应（17例）	*P*值
生命体征			
体温≥38°C伴寒战	9（60.0）	9（52.9）	0.735
收缩压<90mmHg	2（13.3）	0（0.0）	0.212
需要吸氧维持SaO_2_>90％	2（13.3）	3（17.6）	1.000
各系统不良反应			
心血管系统			
心动过速	4（26.7）	6（35.3）	0.712
心律不齐	2（13.3）	1（5.9）	0.589
心脏传导阻滞	1（6.7）	1（5.9）	1.000
呼吸系统			
咳嗽	5（33.3）	6（35.3）	1.000
呼吸急促	2（13.3）	1（5.9）	0.589
肺水肿	2（13.3）	1（5.9）	0.589
消化系统			
恶心	9（60.0）	7（41.2）	0.479
呕吐	3（20.0）	3（17.6）	1.000
腹泻	2（13.3）	5（29.4）	0.402
转氨酶增高	5（33.3）	7（41.2）	0.726
胆红素增高	3（20.0）	5（29.4）	0.691
泌尿系统			
急性肾功能不全	2（13.3）	1（5.9）	0.589
尿量减少、水肿	4（26.7）	4（23.5）	1.000
中枢神经系统			
头痛	8（53.3）	7（41.2）	0.723
血液系统			
3～4级中性粒细胞减少	9（60.0）	10（58.8）	1.000
3～4级贫血	5（33.3）	4（23.5）	0.699
3～4级血小板减少	6（40.0）	4（23.5）	0.450

五、CD19 CAR-T细胞治疗中肿瘤局部反应发生情况

肿瘤局部反应发生于CD19 CAR-T细胞输注后5～7 d，其时间与发热等全身症状出现时间一致、或迟于全身症状1～2 d。共15例（46.9％）患者发生肿瘤局部反应，其中CR 9例、PR 5例、SD 1例。有效组14例（60.9％）出现肿瘤局部反应，1～2级CRS 6例（42.9％），3～4级CRS 8例（57.1％）；SD+PD组1例（11.1％）出现肿瘤局部反应，其CRS为1级；两组肿瘤局部反应率差异有统计学意义（*P*＝0.018）。

六、CD19 CAR-T细胞体内扩增情况

1. 外周血CD19 CAR-T细胞扩增情况：23例有效患者CD19 CAR-T细胞峰值为16.8％（5.3％～48.2％），高于SD + PD组患者的2.9％（1.5％～5.7％），差异有统计学意义（*z*＝−4.297，*P*<0.001）。两组峰值均出现在CD19 CAR-T细胞输注后第7～14天，在输注14 d以后逐渐下降。

23例有效患者中，14例出现肿瘤局部反应，其CD19 CAR-T细胞峰值为22.2％（10.5％～48.2％），高于9例未出现肿瘤局部反应患者的12.6％（5.3％～21.6％），差异有统计学意义（*z*＝−3.213，*P*＝0.001）。将出现肿瘤局部反应的所有15例患者，分为多发肿块组（10例）和单发肿块组（5例），多发肿块组CD19 CAR-T细胞峰值为35.8％（1.5％～48.2％），高于单发肿块组的16.8％（10.5％～18.5％），差异有统计学意义（*z*＝−2.023，*P*＝0.040）。

2. 病灶局部CD19 CAR-T细胞扩增情况：15例患者出现肿瘤局部反应，其中7例引流可以获得腹腔积液、胸腔积液，引流液流式检测CD19 CAR-T细胞比例为4.0％（1.5％～13.5％）。1例腿型淋巴瘤患者（例6）CD19 CAR-T细胞治疗后局部肿胀明显伴疼痛，切开引流大量暗红色液体，CD19 CAR-T细胞比例为2.25％。1例肝脏淋巴瘤患者（例2）CD19 CAR-T细胞治疗中腹胀明显，腹部CT提示肝脏水肿，肝穿活组织病理检查提示非酒精性脂肪性肝病，CD3散在阳性，肝组织CD19 CAR-T细胞比例为2.83％。另外6例患者均为颈部、腋下及腹股沟等浅表肿瘤的局部肿胀、压迫症状，因取材困难未获得标本。

七、有效患者CD19 CAR DNA表达水平变化

23例有效患者中，有局部反应者CD19 CAR DNA表达水平更高，与无局部反应患者比较差异有统计学意义（*z*＝−2.709，*P*＝0.007）。在CD19 CAR-T细胞治疗60 d后，有局部反应的患者CD19 CAR DNA表达下降缓慢，而无局部反应的患者CD19 CAR DNA表达下降迅速。

八、CD19 CAR-T细胞治疗中细胞因子分泌变化

各细胞因子在输注CD19 CAR-T细胞后第4～14天达峰值，14 d以后逐渐下降。各细胞因子峰值：IL-6为34.6（16.2～88.4）ng/L，TNF-α为21.9（15.9～29.4）ng/L，IL-2R为4402（3350～6500）U/ml，IL-10为33.1（14.6～55.2）ng/L。IL-6及TNF-α峰值水平有效组高于SD+PD组，差异有统计学意义（*z*值分别为−3.835、−3.815，*P*值均<0.001）。

九、疗效出现时间

获得ORR的且出现肿瘤局部反应的14例患者，其CD19 CAR-T细胞峰值出现时间为9（6～12）d；IL-6峰值出现时间为10（6～14）d；肿瘤局部反应出现时间为9（7～15）d；肿瘤瘤体开始缩小的时间为14（11～22）d。肿瘤局部反应出现和瘤体缩小时间，均迟于全身不良反应开始的时间。

十、预后分析

中位随访13（4～26）个月，有效组1年OS率为（54.5±13.4）％，中位OS时间未达到；SD+PD组1年OS率为（11.1±10.5）％，中位OS时间4（95％ *CI* 3.026～4.974）个月（*P*<0.001）（图1A）。23例有效患者中，14例出现肿瘤局部反应的患者，1年OS率为（75.0±12.7）％，中位OS时间未达到；9例未出现肿瘤局部反应的患者1年OS率为（34.6±18.3）％，中位OS时间12（95％ *CI* 3.677～20.323）个月（*P*＝0.169）（图1B）。

**图1:**
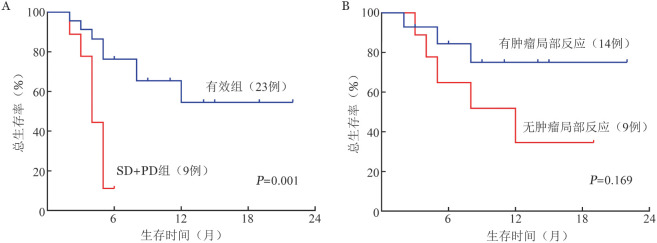
CD19 CAR-T细胞治疗后复发/难治B细胞非霍奇金淋巴瘤患者生存曲线 CAR-T：嵌合抗原受体T细胞；SD：疾病稳定；PD：疾病进展。A：有效组与SD+PD组比较；B：有无肿瘤局部反应比较

## 讨论

CD19 CAR-T细胞治疗在R/R急性B淋巴细胞白血病（B-ALL）和R/R NHL患者中应用越来越广泛[9]–[10]，治疗相关的不良反应也逐步被认识并报道，包括CRS、CRES、B细胞耗竭、血细胞减少等[11]。但上述不良反应的分级及临床处理手段大多参照CD19 CAR-T细胞治疗复发/难治B-ALL的经验，多以全身反应为主。而淋巴瘤兼具实体肿瘤的特点，因此治疗相关的不良反应除全身反应外，亦会出现肿瘤局部反应[12]。本文32例应用CD19 CAR-T细胞且病灶>7.5 cm的R/R NHL患者中，15例治疗期间出现肿瘤局部反应。

CD19 CAR-T细胞治疗过程中可以通过多种机制引起组织器官毒性。首先，CAR-T细胞靶向的肿瘤相关抗原如果亦表达在正常组织或细胞上，这些组织或细胞可能会被破坏，引起相应毒性，例如正常的B淋巴细胞被抗CD19 CAR-T细胞消耗导致B细胞耗竭[13]–[15]。其次，CAR-T细胞亦可能通过与肿瘤细胞表面不表达的蛋白质发生意外交叉反应而损伤正常组织[16]–[17]。CAR-T细胞与肿瘤接触而被激活，导致靶细胞的裂解进而释放炎性细胞因子[18]–[21]，CRS的发展与之密切相关，其发生率和严重程度与体内CAR-T细胞激活和扩增程度、肿瘤负荷相关[22]–[23]。再次，CAR-T细胞治疗中，另外一个常见不良反应是肿瘤溶解综合征（TLS），淋巴瘤广泛骨髓浸润或肿瘤体积较大者易发生TLS[24]。CAR-T细胞在较大瘤体肿瘤局部的扩增、局部出现的TLS均为发生肿瘤局部反应的可能原因。最后，临床试验选择的CAR-T细胞，通常含有来源于小鼠单克隆抗体的抗原识别域，由于外来蛋白的免疫原性，CAR-T细胞治疗过程中可能出现细胞和体液免疫排斥反应[25]–[27]，宿主对外来蛋白的免疫原性识别，从而导致速发型过敏反应，有文献证实了不良反应发生后，患者血清中存在人抗鼠抗体以及血清胰酶升高的现象，支持了不良反应的发生与该速发型过敏反应相关的观点[28]。本组患者均为病灶>7.5 cm的复发/难治NHL患者，存在治疗过程中出现上述明显不良反应的可能性。

CAR-T细胞治疗复发/难治NHL过程中，肿瘤局部反应可能与上述原因导致的炎性反应有关[29]，本组病例观察到的局部反应包括局部红肿热痛、瘤体因炎性反应迅速增大造成局部压迫、局部炎性渗出等。红肿热痛多出现在浅表部位的瘤体，而瘤体增大除浅表部位外亦多见于纵隔、腹膜后等深部淋巴瘤瘤体，局部炎性渗出多出现在临近浆膜腔部位的瘤体，如胸腔、腹腔等。

对于CAR-T治疗过程中全身反应的诊断和治疗，目前已有相关指南，但既往关于肿瘤局部反应的报道多为个案[30]，关于局部反应的诊断和治疗尚无共识。本文15例发生局部反应的复发/难治NHL局部处理，包括对症镇痛、局部切开和（或）穿刺引流，对于有威胁生命的局部压迫或梗阻等情况，予全身应用糖皮质激素或IL-6抗体治疗。虽然差异无统计学意义，但我们观察到有效组中出现局部反应者1年OS率高于未出现局部反应者（75.0％对34.6％，*P*＝0.169），而且其CAR-T扩增也明显高于未出现局部反应者；同时，我们在出现局部反应的患者的引流液中发现CAR-T细胞的扩增。这是否提示CAR-T细胞可能具有向肿瘤局部驱化的能力，尚需要在后续的研究中进一步证实。
